# VEXAS and Myelodysplastic Syndrome: An Interdisciplinary Challenge

**DOI:** 10.3390/jcm13041049

**Published:** 2024-02-12

**Authors:** Virginie Kreutzinger, Anne Pankow, Zhivana Boyadzhieva, Udo Schneider, Katharina Ziegeler, Lars Uwe Stephan, Jan Carl Kübke, Sebastian Schröder, Christian Oberender, Philipp le Coutre, Sebastian Stintzing, Ivan Jelas

**Affiliations:** 1 Department of Radiology, Vivantes Klinikum im Friedrichshain, 10249 Berlin, Germany; 2 Department of Rheumatology and Clinical Immunology, Campus Charité Mitte, Charitéplatz 1, 10117 Berlin, Germany; 3 Department of Hematology, Oncology, and Cancer Immunology, Campus Charité Mitte, Charitéplatz 1, 10117 Berlin, Germany

**Keywords:** autoinflammation, imaging, myelodysplastic syndrome, VEXAS syndrome

## Abstract

VEXAS (vacuoles, E1 enzyme, X-linked, autoinflammatory, somatic) syndrome is a recently recognized systemic autoinflammatory disease caused by somatic mutations in hematopoietic progenitor cells. This case series of four patients with VEXAS syndrome and comorbid myelodysplastic syndrome (MDS) aims to describe clinical, imaging, and hematologic disease presentations as well as response to therapy. Four patients with VEXAS syndrome and MDS are described. A detailed analysis of imaging features, hemato-oncological presentation including bone marrow microscopy and clinical–rheumatological disease features and treatment outcomes is given. All patients were male; ages ranged between 64 and 81 years; all were diagnosed with MDS. CT imaging was available for three patients, all of whom exhibited pulmonary infiltrates of varying severity, resembling COVID-19 or hypersensitivity pneumonitis without traces of scarring. Bone marrow microscopy showed maturation-disordered erythropoiesis and pathognomonic vacuolation. Somatic mutation in the *UBA1* codon 41 were found in all patients by next-generation sequencing. Therapy regimes included glucocorticoids, JAK1/2-inhibitors, nucleoside analogues, as well as IL-1 and IL-6 receptor antagonists. No fatalities occurred (observation period from symptom onset: 18–68 months). Given the potential underreporting of VEXAS syndrome, we highly recommend contemporary screening for *UBA1* mutations in patients presenting with ambiguous signs of systemic autoinflammatory symptoms which persist over 18 months despite treatment. The emergence of cytopenia, especially macrocytic hyperchromic anemia, should prompt early testing for *UBA1* mutations. Notably conspicuous, pulmonary alterations in CT imaging of patients with therapy-resistant systemic autoinflammatory symptoms should be discussed in interdisciplinary medical teams (Rheumatology, Hematology, Radiology and further specialist departments) to facilitate timely diagnosis during the clinical course of the disease.

## 1. Introduction

The VEXAS syndrome is an autoinflammatory systemic disease first described in 2020 [1]; the acronym stands for vacuoles, E1 enzyme, X-linked, autoinflammation and somatic (mutation). The majority of affected patients are men in their fifties to seventies, presenting with a severe inflammatory syndrome and hematologic abnormalities [2,3,4]. Diagnosis is made by confirmation of alterations in the *UBA1* gene, which is typically performed using genomic DNA from peripheral blood leukocytes or bone marrow tissue [5,6,7]. The disease is characterized by a somatically acquired mutation affecting methionine 41 of the E1-ubiquitin ligase *UBA1*, resulting in the expression of a catalytically impaired isoform that drives inflammation processes in the body. Common clinical features observed in VEXAS syndrome patients include skin lesions (83%), non-infectious fever (64%), weight loss (62%), lung involvement (50%), ocular symptoms (39%), relapsing chondritis (36%), venous thrombosis (35%), lymphadenopathy (34%), and arthralgia (27%) [3]. Additionally, patients with VEXAS syndrome experience progressive hematologic abnormalities such as macrocytic anemia, thrombocytopenia, and myeloid dyspoiesis [8,9,10]. In a substantial number of cases, the disease may progress to an overt hematologic malignant condition [11,12,13]. Bone marrow aspirates typically show characteristic vacuoles restricted to myeloid and erythroid precursor cells [6,9]. Due to the novelty of the disease, there are currently no evidence-based recommendations for treatment. Treatment options include the use of glucocorticoids, conventional disease-modifying antirheumatic drugs (DMARDs), biologically targeted drugs, and allogeneic hematopoietic stem cell transplantation [14,15,16,17]. The literature reflects the difficulty of diagnosing VEXAS syndrome patients during their lifetime with many publications being retrospective in nature.

Our case series aims to demonstrate that the diagnosis of VEXAS syndrome can be made during the course of the illness if clinical characteristics are carefully documented, discussed in an interdisciplinary manner, and if patients receive the necessary diagnostic evaluations. Furthermore, we illustrate the management and response to therapy in four patients with VEXAS syndrome who also have concurrent MDS, and we explain why a comprehensive investigation of the disease’s imaging features could be helpful in identifying these very rare patients.

## 2. Materials and Methods

### 2.1. Imaging

All available imaging datasets were reviewed and discussed in consensus by two radiologists with deep clinical expertise in rheumatological imaging (K.Z. and V.K.). Special consideration was given to previously described imaging features of VEXAS syndrome, including pleuro-pulmonary manifestations, polychondritis, and lymphadenopathy as well as signs of vasculitis. Special consideration was given to imaging manifestations in the skeletal system including bone marrow changes associated with MDS and patterns of arthritis, which could not be attributed to typical aging.

### 2.2. Bone Marrow Smear

Approximately 5 mL of semi-liquid bone marrow content was immediately processed on pre-prepared glass slides and stained with Wright–Giemsa and iron staining.

### 2.3. Next-Generation-Sequencing

Genomic DNA was extracted from bone marrow (*n* = 4) and samples were analyzed by next-generation sequencing using a 68- and a 98-gene panel to detect mutations of *UBA1* and further somatic mutations (e.g., *U2AF1*, *DNMT3A* and *TET2*).

### 2.4. Ethical Approval, Data Availability and Patient and Public Involvement

All patients provided written informed consent for the use of their data for scientific purposes. This investigation was overseen by the ethics committee of Charité Universitätsmedizin Berlin (EA4/053/21). All data and materials from this study are available upon reasonable request to the corresponding author. There was no specific patient or public involvement in this investigation.

## 3. Results

### 3.1. Clinical Features, Treatment and Outcome

Patient 1: A male patient in his late 60 s presented with a history of peripheral deep vein thromboses, dyspnea, muscle weakness, Raynaud-like symptoms, and persistent fever. The patient was over 35 months under rheumatological outpatient and inpatient care and received during this time DMARDs (methotrexate, leflunomide) and glucocorticoids intermittently with more than 20 mg prednisolone per day. Over time, the patient developed persistent macrocytic hyperchromic anemia, which could not be explained by a deficiency in folic acid, vitamin B12, side effects of present medication, or other diagnosed diseases. The patient was referred to the divisions of rheumatology and hematology at Charité—Universitätsmedizin Berlin for further care. After a comprehensive interdisciplinary evaluation of the patient’s clinical course so far, an investigation into macrocytic hyperchromic anemia was initiated, ultimately leading to a bone marrow aspiration. The bone marrow aspirate smears from the patient demonstrated signs of dysplasia, including nuclear abnormalities in erythroid precursor cells and multinucleated micro-megakaryocytes. Furthermore, cytoplasmic vacuolation of myeloid and erythroid precursors was particularly noticeable during the cytological examination. Next-generation sequencing (NGS) detected mutations in DNMT3A and UBA1, leading to a diagnosis of low-risk MDS (IPSS-R of 2) and VEXAS syndrome.

Due to the mild cytopenia, a “watch and wait” strategy was initially pursued for MDS, since symptoms of autoinflammation persisted and led to an overall deterioration in his health. Due to the limited data available on treatment options in VEXAS syndrome, an off-label use of the JAK1/2 inhibitor ruxolitinib was initiated at 20 mg twice daily, and glucocorticoids were discontinued [18].

During the treatment, the dose of ruxolitinib was adjusted due to side effects such as dizziness, headache, fever, and constipation. The first dose reduction in ruxolitinib was to 15 mg, which was followed by adjustments to 10 mg and finally to 5 mg twice daily. Additionally, despite these those adjustments, muscle weakness and fever persisted, leading to the initiation of concurrent treatment with glucocorticoids (15 mg Prednisolon once daily).

Simultaneously, blood tests over six months consistently indicated a worsening of anemia. Consequently, a repeat bone marrow aspiration was performed, revealing a progressive maturation disorder, particularly in erythropoiesis, while the blast cell count remained normal. In the context of persistently limited data regarding further alternative treatment options, we decided to initiate a treatment with 5-azacytidine (75 mg/m^2^ daily for 7 days, followed by a rest period of 21 days) [14].

After the switch to 5-azacytidine, rapid improvement in autoinflammatory symptoms and anemia was observed. However, continued treatment with 7.5 mg of prednisolone once daily was necessary to control clinical symptoms like muscle weakness and fever. The patient received six cycles of 5-azacytidine (28-day treatment cycle). The tolerability of the therapy over the six cycles was good. Clinically relevant signs of toxicity were not observed. As of now, blood results and autoinflammatory symptoms have further stabilized, but long-term effects are yet to be determined and need to be followed up closely

Patient 2: A male in his mid-70s presented with recurrent episodes of fever, dyspnea, and unexplained pulmonary inflammation (no detected pathogen, no elevation of procalcitonin), one episode of pleuritis, recurrent sterile parotitis, and deep venous thrombosis. Furthermore, he experienced arthralgia in the wrists and had elevated rheumatoid factors. Imaging findings were suggestive of interstitial lung disease, with peripheral consolidations and mild ground glass opacities prompted pronchoalveolar lavage, which revealed a CD4/CD8 ratio of 0.9; antigen testing for hypersensitivity pneumonitis (which was initially suspected) was negative. Pulmonary symptoms responded to prednisolone, but withdrawal attempts resulted in a recurrence of dyspnea, requiring a continued dose of 20 mg prednisolone. Insufficient control of autoinflammatory symptoms over 26 months and worsening of hematopoiesis over 4 months prompted the transfer of the patient to the divisions of rheumatology and hematology at Charité—Universitätsmedizin Berlin for further diagnostics and clinical care. Following a thorough interdisciplinary assessment in both divisions, considering the patient’s clinical history and the progressing inefficacy of hematopoiesis, a bone marrow biopsy was performed.

In the bone marrow aspirate smears of the patient, signs of dysplasia and cytoplasmic vacuolation of myeloid and erythroid precursors were detected. NGS testing revealed mutations in TET2, U2AF1, and UBA1, leading to the diagnosis of low-risk MDS (IPSS-R of 2.5) and VEXAS syndrome.

The initiated treatment with prednisolone (20 mg) was quickly escalated to 5-azacytidine due to persistent anemia and thrombocytopenia. Under the combination treatment of prednisolone and 5-azacytidine, complete recovery of blood cell counts was achieved, but only limited improvement in autoinflammation (fever) was observed. A dose reduction in prednisolone resulted in an immediate recurrence of severe arthralgia and more frequent fever episodes. Therefore, we transitioned to ruxolitinib (JAK1/2 inhibitor) at a dose of 20 mg twice daily.

With ruxolitinib, there was a slight decrease in hemoglobin levels, while platelet and leukocyte counts remained stable. However, there was a significant improvement in autoinflammatory symptoms (no episodes of fever and/or arthralgia) and also a substantial clinical improvement in pulmonary symptoms as well as pulmonary imaging findings. The tolerability was excellent, and no side effects occurred, allowing ruxolitinib therapy to continue without complications for 12 months up to the current point. However, the long-term effects remain undetermined, and the patient is currently under close medical care and follow-up.

Patient 3: A male in his early 80s presented with intermittent fever, unintentional weight loss, and a history of skin rashes and polyarthritis. Biopsy of affected skin showed neutrophilic dermatosis, raising suspicion of a paraneoplastic origin. Based on imaging, a diagnosis of polyarticular CPPD was made, and treatment with systemic prednisolone and an interleukin-1 antagonist (anakinra, starting dosage 2 mg/kg daily) was initiated but with limited clinical response. The patient underwent rheumatological outpatient and inpatient care for over 18 months, since the patients develop gradual pancytopenia (macrocytic hyperchromic anemia) referral to the divisions of hematology at Charité—Universitätsmedizin Berlin for further diagnostics. After intensive interdisciplinary examination of the patient’s case by the departments of Hematology and Rheumatology, due to a suspected diagnosis of MDS and VEXAS syndrome, a bone marrow biopsy was performed. The bone marrow analysis revealed hypercellularity with increases in two lineages. Furthermore, there were prominent signs of dysplasia, megaloblastoid changes, and nuclear abnormalities in erythroid precursor cells along with typical cytoplasmic vacuolation in myeloid and erythroid precursor cells. NGS testing did not reveal any MDS-specific mutations but did identify a UBA1 mutation. Consequently, the patient was diagnosed with very-low-risk MDS (IPSS-R of 1) and VEXAS syndrome.

Due to ongoing clinical signs of autoinflammation, an off-label treatment with ruxolitinib (20 mg twice daily) in combination with prednisolone (20 mg) was initiated. Repeated attempts to taper off prednisolone failed during the course of treatment because, at a dose below 5 mg, the autoinflammatory symptoms, especially arthritis, worsened significantly. The combination therapy was well-tolerated, and there were no significant toxicities or side effects that necessitated a modification of the treatment with ruxolitinib. Blood counts, especially anemia, recovered quickly after initiating the treatment. The patient is presently receiving attentive medical supervision and ongoing follow-up.

Patient 4: A male patient in his mid-60s presented with intermittent fever, night sweats, and a history of dermatosis and chronic polyarthritis. Chronic neutrophilic urticarial dermatosis and adult-onset Still’s disease were suspected. Sequential treatments with DMARD (methotrexate), anakinra (an interleukin-1 antagonist), and canakinumab (an interleukin-1β blocker) were attempted but terminated due to suspicion of therapy-induced pancytopenia and interstitial lung disease.

Due to inadequate control of autoinflammatory symptoms over 68 months, along with an existing and progressively worsening macrocytic hyperchromic anemia, as well as mild thrombocytopenia, the patient sought a second opinion at Charité—Universitätsmedizin Berlin for further diagnostics and clinical care. Following an interdisciplinary presentation and discussion of the case, a suspected diagnosis of MDS and VEXAS Syndrome was made. An extended hematological–oncological diagnostic evaluation was initiated. Subsequently, a bone marrow biopsy was performed, revealing binuclearity in erythroid cell precursors as well as noticeable cytoplasmic vacuolation of myeloid and erythroid precursors. No pathological increase in blast cells was observed. Subsequent NGS examination identified a mutation in the EZH2 gene with unclear significance and a UBA1 mutation, leading to a diagnosis of very low-risk MDS (IPSS-R of 1) and VEXAS syndrome.

Therapy with 5-azacytidine (daily for 7 days, followed by a rest period of 21 days) in combination with prednisolone (7.5 mg) was initiated. Blood improved over time, particularly the macrocytic hyperchromic anemia, as did the clinical signs of autoinflammation. Prednisolone was completely discontinued, and the therapy with 5-azacytidine was dose-reduced from 7 to 4 days per cycle (28-day treatment cycle) starting from cycle 16. Cumulatively, the patient received 30 cycles of 5-azacytidine with clinically well-controlled VEXAS syndrome and hematologically stable MDS. The patient is currently undergoing treatment with 5-azacytidine and is under careful medical monitoring.

Further patient characteristics are given in Table 1 and Table 2

### 3.2. Imaging

#### 3.2.1. Chest

**Figure 1:**
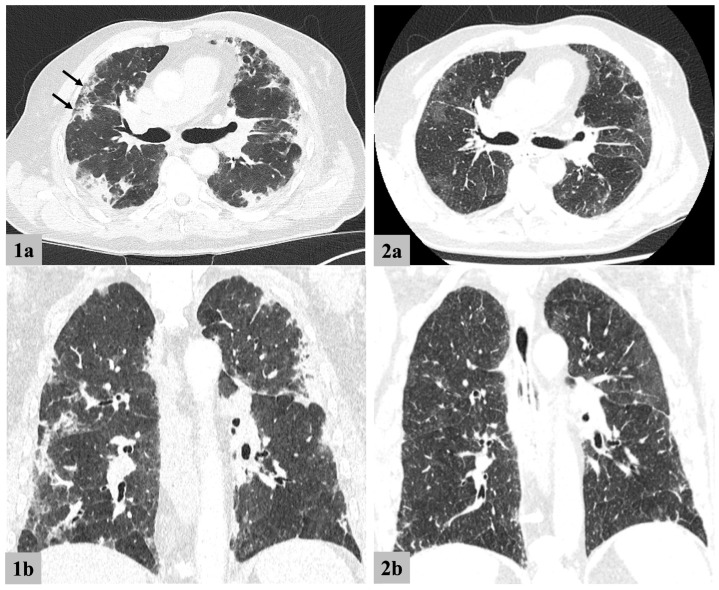
Pulmonary findings on computed tomography (Patient 2). 1 = time of initial diagnosis; 2 = two months later. a = axial reconstructions; b = coronal reconstructions. Marked subpleural consolidations without topographical predominance are seen (marked with arrows). After two months, patchy ground glass opacities remain without notable scarring.

Three of the patients received CT imaging of the lungs at different time points. In all these patients, a pattern of almost exclusively peripherally located consolidations, without upper- or lower lobe predominance was observed—one of the cases (patient 2) is depicted in Figure 1. In this patient, consolidations resolved over a course of two months with still clearly visible ground glass opacities in areas of previous consolidations yet no evident fibrosis or scarring. Hilar lymphadenopathy was seen in two out of four (patients 2 and 3) patients; both symmetric and asymmetric manifestations were seen in the same patient (patient 2) over the course of the disease. No bronchial wall abnormalities indicating relapsing chondritis were observed.

#### 3.2.2. Joints and Bones

Spinal imaging was only available for two patients, one of whom exhibited extensive bone marrow reconversion, which was consistent with MDS. Two patients (2 and 3) reported symmetric arthralgia of the wrists, prompting imaging investigations of arthritis. One patient received MR imaging, showing non-specific patchy bone marrow edema of the carpalia without synovitis, erosion, or tendon involvement. Patient 3 received a whole-body PET-CT which showed symmetrically increased tracer uptake in both wrists; in this patient, radiography showed degenerative lesions and calcifications of the triangular fibrocartilage of the wrists, but no erosions, resulting the diagnosis of CPPD.

### 3.3. Bone Marrow Microscopy

**Figure 2:**
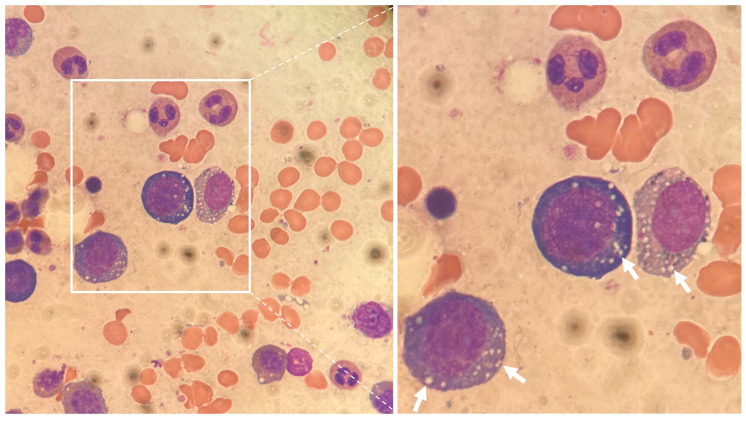
Bone marrow aspirate smear (Patient 1). Left: microscopic magnification 100×. Characteristic cytoplasmic vacuolation of myeloid and erythroid precursors is indicated by white arrows.

All patients exhibited an increase in cellularity (ranging between 50% and 80%) with maturation-disordered erythropoiesis (megaloblastic precursors, binucleation, nuclear rounding) and pathognomonic vacuolation (Figure 2)—the latter in three out of four patients.

The granulopoiesis to erythropoiesis (G:E) ratio shifted in favor of granulopoiesis to ratios of 3–6:1. No pathological increases in blasts were observed. Next-generation sequencing (NGS) revealed somatic mutations in the UBA1 codon in all patients—results are given in Table 2.

## 4. Discussion

In this report, we present a detailed account of four patients diagnosed with VEXAS syndrome and comorbid MDS from a single center in Germany (Charité—Universitätsmedizin Berlin). All patients exhibited systemic autoinflammatory symptoms that progressed over an extended time period (18 to 68 months). None of the four patients showed significant responsiveness to various immunosuppressive treatments throughout their clinical course, having either no response or only partial responsiveness. Despite receiving outpatient and intermittently inpatient rheumatological care, a definitive diagnosis could not be established based on their clinical progression.

The decision to refer these patients to our specialized clinical departments was prompted by uncontrolled systemic autoinflammatory symptoms and discrete worsening of blood counts, particularly progressive macrocytic hyperchromic anemia. The comprehensive interdisciplinary evaluation of these patients, involving the Departments of Rheumatology, Hematology, and Radiology, led to the diagnosis of MDS-VEXAS syndrome within six weeks after the initial consultation. Based on our observations and experiences, the prolonged time to diagnose represents one of the main challenges for MDS-VEXAS syndrome patients. This fact is underscored but not prominently highlighted in the existing literature. In addition to the absence of evidence-based treatment options, the extended time to diagnosis is, from our perspective, one of the key medical needs that should be addressed in the future. This becomes particularly evident when considering the findings of Huang et al., who reported in their series that 10 out of 25 patients died from disease-related causes such as progressive anemia or therapy-related complications [5]. The delayed diagnosis also explains why many comprehensive reports are of a retrospective nature [1,2].

Therefore, we suggest that a basic standardized diagnostic criteria classifier should be developed to simplify the identification of these patients in clinical routine. This MDS-VEXAS syndrome classifier should include patient characteristics, key clinical features, the number of prior therapies, duration of symptoms, laboratory findings, and radiological alterations.

The clinical courses of the four reported MDS-VEXAS syndrome patients reflect the current medical situation in routine patient care and align with previously published series [1,2,5]. Patients with VEXAS syndrome have a lengthy medical history, spanning months to years, characterized by ambiguous autoinflammatory symptoms.

The clinical phenotype and autoinflammatory symptoms of patients with VEXAS syndrome, with or without MDS, have already been well described by other working groups in the literature [1,2,5,19]. Our four patients showed no new clinical features and insights. All of our patients presented with systemic autoinflammatory symptoms, especially fever, which was in line with publications by Beck et al. and Georgin-Lavialle. Arthralgia and arthritis were observed in three out of four patients, which was consistent with previous descriptions in the literature [2,20]. The chest images of our MDS-VEXAS syndrome patients demonstrated lung involvement, as previously described in the literature. The radiologically identified consolidations transitioning to ground glass opacity, with no discernible parenchymal scarring, are consistent with previous publications [6,21,22,23]. Notably, there was a radiological overlap with COVID-19, which was characterized by predominantly peripheral/subpleural infiltrates. Interestingly, this manifested in a reversed order compared to early COVID-19, where ground glass opacity is typically seen [7]. As described for patient 2, these imaging findings have limited specificity and show substantial overlap to interstitial lung diseases; thus, a radiologist with limited clinical background information may misdirect clinical diagnosis, which further highlights the need for a truly interdisciplinary approach. Imaging findings in MDS, especially the loss of the typical fat signal of bone marrow in T1-weighted sequences, should not be mistaken for bone marrow edema. In such investigations, supplying the diagnosing radiologist with information about comorbid MDS, rather than just inquiring about the presence of arthritis or other inflammatory skeletal findings, will safeguard against overdiagnosis of arthritis. One of our patients was diagnosed with CPPD on imaging; the interplay of systemic autoinflammation and symptomatic crystal deposition disease may be of interest in future research. Overall, radiological findings must be considered in an interdisciplinary context, alongside other clinical parameters and symptoms, and could be implemented in the basic standardized diagnostic criteria classifier mentioned above.

Limited but promising results have been achieved with the JAK1/2 inhibitor ruxolitinib or the hypomethylating agent 5-azacytidine [8,9,10,18,24]. Due to this fact and the absence of evidence-based recommendations, we treated our patients with these two substances.

Fortunately, all four of our patients responded to the selected systemic therapy. However, in the cases of patients 1 and 2, insufficient clinical and laboratory therapeutic responses were initially observed, leading to a change in treatment. The mechanistic reasons for treatment response or the lack of response to ruxolitinib or 5-azacytidine in some VEXAS syndrome and MDS patients are currently unclear and cannot be explained by previously published works [8,9,10,14,18,24]

Despite ruxolitinib or 5-azacytidine demonstrating a good treatment response, all four of our patients required intermittent or permanent high doses of systemic glucocorticoids (7.5–50 mg/day prednisolone). This fact underscores the clinical need to develop further targeted therapy options for the autoinflammatory spectrum of the disease. In particular, the JAK1/2 inhibitor ruxolitinib appears to be a suitable candidate for reducing the concurrent need for glucocorticoids in VEXAS syndrome patients, whether or not they have myeloid neoplasia [9].

Our clinical experience supports the effectiveness of ruxolitinib and 5-azacytidine, as described in the literature. Furthermore, we see the potential for both agents to be used in combination treatment for MDS-VEXAS syndrome and MDS patients. Phase II data of ruxolitinib in combination with 5-azacytidine in myelodysplastic syndrome/myeloproliferative neoplasms demonstrated a tolerable safety profile with a good response rate [21]. Therefore, a prospective study will be required to explore the potential of this combination in the future.

The only known treatment that leads to a cure for MDS-VEXAS syndrome patients is allogeneic hematopoietic stem cell transplantation (AHSCT). Currently, an ongoing phase II study for patients with VEXAS syndrome aims to evaluate the impact of AHSCT for this condition (NCT05027945)

In summary, our report emphasizes the challenges posed by overlapping symptoms in rheumatological and hematological conditions, compounded by the rarity of the disease, making it difficult to identify these patients in routine clinical care. Based on our clinical observations and experience, this study identifies prolonged diagnosis as one of the main problems for MDS-VEXAS syndrome patients. Therefore, we see an opportunity for the development of a basic standardized diagnostic criteria classifier for clinical routine to prevent delayed diagnosis in their clinical course. This future MDS-VEXAS syndrome classifier should include patient characteristics, key clinical features, the number of prior therapies, duration of symptoms, laboratory findings, and radiological alterations. Moreover, we recommend that patients with prolonged therapy-resistant autoinflammatory symptoms undergo a comprehensive interdisciplinary evaluation and discussion. This should involve at least the Departments of Rheumatology, Hematology, and Radiology. Only an early and accurate diagnosis, including molecular genetic analyses, allows for the early treatment of these patients and their inclusion in current and future clinical studies.

## Figures and Tables

**Table 1:** Laboratory findings and observation periods. Values upon initial presentation at our center. * = both patients received high-dose oral prednisolone (>50 mg/d). Observation periods: from first patient reported systemic autoinflammatory symptoms until time of manuscript preparation.

	Patient 1	Patient 2	Patient 3	Patient 4
Hemoglobin (g/dL)	10.3	8.2	8.1	8.0
Mean corpuscular volume (fL)	109.1	124.1	95.0	111.0
White blood count (/nL)	6.54	6.60	6.67	4.70
Platelet count (/nL)	195	38	212	82
C-reactive protein at initial presentation (mg/L)	98.3	26.5 *	139.3	10.5 *
Observation period (months)	35	26	18	68

**Table 2:** Next-generation sequencing.

	UBA1	Further Somatic Mutations
**Patient 1**	*UBA1* codon 41 (p.Met41Val (NM_003334.4:c.121A>G))	*DNMT3A* (p.Arg882Cys (NM_022552.5:c.2644C>T))
**Patient 2**	*UBA1* codon 41 (p.Met41Val (NM_003334.4:c.121A>G))	*TET2* (p.M633I (c.1899G>A)), *U2AF1* (p.Q157P (c.470A>C))
**Patient 3**	*UBA1* codon 41 (p.Met41Leu (NM_003334.4:c.121A>C))	none
**Patient 4**	*UBA1* codon 41 (p.Met41Leu (NM_003334.4:c.121A>C))	*EZH2* gene: intronic, non-coding region 2-base deletion near a splice site, with unclear significance

## Data Availability

All data and materials presented in this study are available on reasonable request from the corresponding author upon reasonable request.

## References

[1] Beck D.B., Ferrada M.A., Sikora K.A., Ombrello A.K., Collins J.C., Pei W., Balanda N., Ross D.L., Ospina Cardona D., Wu Z. (2020). Somatic Mutations in UBA1 and Severe Adult-Onset Autoinflammatory Disease. N. Engl. J. Med..

[2] Georgin-Lavialle S., Terrier B., Guedon A.F., Heiblig M., Comont T., Lazaro E., Lacombe V., Terriou L., Ardois S., Bouaziz J.D. (2022). Further characterization of clinical and laboratory features in VEXAS syndrome: Large-scale analysis of a multicentre case series of 116 French patients. Br. J. Dermatol..

[3] Koster M.J., Kourelis T., Reichard K.K., Kermani T.A., Beck D.B., Cardona D.O., Samec M.J., Mangaonkar A.A., Begna K.H., Hook C.C. (2021). Clinical Heterogeneity of the VEXAS Syndrome: A Case Series. Mayo Clin. Proc..

[4] Ruffer N., Krusche M. (2023). VEXAS syndrome: A diagnostic puzzle. RMD Open.

[5] Huang H., Zhang W., Cai W., Liu J., Wang H., Qin T., Xu Z., Li B., Qu S., Pan L. (2021). VEXAS syndrome in myelodysplastic syndrome with autoimmune disorder. Exp. Hematol. Oncol..

[6] Patel N., Dulau-Florea A., Calvo K.R. (2021). Characteristic bone marrow findings in patients with UBA1 somatic mutations and VEXAS syndrome. Semin. Hematol..

[7] Poulter J.A., Collins J.C., Cargo C., De Tute R.M., Evans P., Ospina Cardona D., Bowen D.T., Cunnington J.R., Baguley E., Quinn M. (2021). Novel somatic mutations in UBA1 as a cause of VEXAS syndrome. Blood.

[8] Austestad J., Madland T.M., Sandnes M., Haslerud T.M., Benneche A., Reikvam H. (2023). VEXAS Syndrome in a Patient with Myeloproliferative Neoplasia. Case Rep. Hematol..

[9] Cherniawsky H., Friedmann J., Nicolson H., Dehghan N., Stubbins R.J., Foltz L.M., Leitch H.A., Sreenivasan G.M., Ambler K.L.S., Nevill T.J. (2023). VEXAS syndrome: A review of bone marrow aspirate and biopsies reporting myeloid and erythroid precursor vacuolation. Eur. J. Haematol..

[10] Himmelmann A., Brucker R. (2021). The VEXAS Syndrome: Uncontrolled Inflammation and Macrocytic Anaemia in a 77-Year-Old Male Patient. Eur. J. Case Rep. Intern. Med..

[11] Lotscher F., Seitz L., Simeunovic H., Sarbu A.C., Porret N.A., Feldmeyer L., Borradori L., Bonadies N., Maurer B. (2021). Case Report: Genetic Double Strike: VEXAS and TET2-Positive Myelodysplastic Syndrome in a Patient with Long-Standing Refractory Autoinflammatory Disease. Front. Immunol..

[12] Obiorah I.E., Patel B.A., Groarke E.M., Wang W., Trick M., Ombrello A.K., Ferrada M.A., Wu Z., Gutierrez-Rodrigues F., Lotter J. (2021). Benign and malignant hematologic manifestations in patients with VEXAS syndrome due to somatic mutations in UBA1. Blood Adv..

[13] Oganesyan A., Hakobyan Y., Terrier B., Georgin-Lavialle S., Mekinian A. (2021). Looking beyond VEXAS: Coexistence of undifferentiated systemic autoinflammatory disease and myelodysplastic syndrome. Semin. Hematol..

[14] Comont T., Heiblig M., Riviere E., Terriou L., Rossignol J., Bouscary D., Rieu V., Le Guenno G., Mathian A., Aouba A. (2022). Azacitidine for patients with Vacuoles, E1 Enzyme, X-linked, Autoinflammatory, Somatic syndrome (VEXAS) and myelodysplastic syndrome: Data from the French VEXAS registry. Br. J. Haematol..

[15] Diarra A., Duployez N., Fournier E., Preudhomme C., Coiteux V., Magro L., Quesnel B., Heiblig M., Sujobert P., Barraco F. (2022). Successful allogeneic hematopoietic stem cell transplantation in patients with VEXAS syndrome: A 2-center experience. Blood Adv..

[16] Kunishita Y., Kirino Y., Tsuchida N., Maeda A., Sato Y., Takase-Minegishi K., Yoshimi R., Nakajima H. (2022). Case Report: Tocilizumab Treatment for VEXAS Syndrome With Relapsing Polychondritis: A Single-Center, 1-Year Longitudinal Observational Study In Japan. Front. Immunol..

[17] Raaijmakers M., Hermans M., Aalbers A., Rijken M., Dalm V., van Daele P., Valk P.J.M. (2021). Azacytidine Treatment for VEXAS Syndrome. Hemasphere.

[18] Heiblig M., Ferrada M.A., Koster M.J., Barba T., Gerfaud-Valentin M., Mekinian A., Coelho H., Fossard G., Barraco F., Galicier L. (2022). Ruxolitinib is more effective than other JAK inhibitors to treat VEXAS syndrome: A retrospective multicenter study. Blood.

[19] Kipfer B., Daikeler T., Kuchen S., Hallal M., Andina N., Allam R., Bonadies N. (2018). Increased cardiovascular comorbidities in patients with myelodysplastic syndromes and chronic myelomonocytic leukemia presenting with systemic inflammatory and autoimmune manifestations. Semin. Hematol..

[20] Ferrada M.A., Sikora K.A., Luo Y., Wells K.V., Patel B., Groarke E.M., Ospina Cardona D., Rominger E., Hoffmann P., Le M.T. (2021). Somatic Mutations in UBA1 Define a Distinct Subset of Relapsing Polychondritis Patients With VEXAS. Arthritis Rheumatol..

[21] Assi R., Kantarjian H.M., Garcia-Manero G., Cortes J.E., Pemmaraju N., Wang X., Nogueras-Gonzalez G., Jabbour E., Bose P., Kadia T. (2018). A phase II trial of ruxolitinib in combination with azacytidine in myelodysplastic syndrome/myeloproliferative neoplasms. Am. J. Hematol..

[22] Kouranloo K., Ashley A., Zhao S.S., Dey M. (2023). Pulmonary manifestations in VEXAS (vacuoles, E1 enzyme, X-linked, autoinflammatory, somatic) syndrome: A systematic review. Rheumatol. Int..

[23] Lohaus N., Schaab J., Schaer D., Balabanov S., Huellner M.W. (2023). VEXAS Syndrome With Tracheal Involvement but Absence of Vasculitis in FDG PET/CT. Clin. Nucl. Med..

[24] Mekinian A., Zhao L.P., Chevret S., Desseaux K., Pascal L., Comont T., Maria A., Peterlin P., Terriou L., D’Aveni Piney M. (2022). A Phase II prospective trial of azacitidine in steroid-dependent or refractory systemic autoimmune/inflammatory disorders and VEXAS syndrome associated with MDS and CMML. Leukemia.

